# Population structure of five native sheep breeds of Sweden estimated with high density SNP genotypes

**DOI:** 10.1186/s12863-020-0827-8

**Published:** 2020-03-06

**Authors:** Christina Marie Rochus, Elisabeth Jonas, Anna M. Johansson

**Affiliations:** 1 Department of Animal Breeding and Genetics; Faculty of Veterinary Medicine and Animal Science, Swedish University of Agricultural Sciences, Box 7023, SE75007 Uppsala, Sweden; 2 UFR Génétique, Élevage et Reproduction, Sciences de la Vie et Santé, AgroParisTech, Université Paris-Saclay, Paris, France; 3 Génétique Physiologie Systèmes d’Elevage (GenPhySE), Animal Genetics Division, INRA, Castanet Tolosan, France; 4 Animal Breeding and Genomics, Wageningen University and Research, Wageningen, the Netherlands

**Keywords:** Sheep, Population structure, North European short-tailed sheep, SNP

## Abstract

**Background:**

Native Swedish sheep breeds are part of the North European short-tailed sheep group; characterized in part by their genetic uniqueness. Our objective was to study the population structure of native Swedish sheep. Five breeds were genotyped using the 600 K SNP array. Dalapäls and Klövsjö sheep are from the middle of Sweden; Gotland and Gute sheep from Gotland, an island in the Baltic Sea; and Fjällnäs sheep from northern Sweden. We studied population structure by: principal component analysis (PCA), cluster-based analysis of admixture, and an estimated population tree.

**Results:**

The analyses of the five Swedish breeds revealed that these breeds are five distinct breeds, while Gute and Gotland are more closely related to each other as seen in all analyses. All breeds had long branch lengths in the population tree indicating they’ve been subjected to drift. We repeated our analyses using 39 K SNP and including 50 K SNP genotypes from other European and southwestern Asian breeds from the Sheep HapMap project and 600 K SNP genotypes from a dataset of French sheep. Results arranged breeds into five groups: south-west Asia, south-west Europe, central Europe, north Europe and north European short-tailed sheep. Within this last group, Norwegian and Icelandic breeds, Finn and Romanov sheep, Scottish breeds, and Gute and Gotland sheep were more closely related while the remaining Swedish breeds and Ouessant sheep were distinct from all breeds and had longer branches in the population tree.

**Conclusions:**

We showed population structure of five Swedish breeds and their structure within European and southwestern Asian breeds. Swedish breeds are unique, distinct breeds that have been subjected to drift but group with other north European short-tailed sheep.

## Background

Sheep (*Ovis aries*) were domesticated 11,000 BP in the Anatolia region [1] from Asian Mouflon (*Ovis orientalis*) [2]. After domestication, sheep spread around the world including spreading into Europe via two main paths, south and north, following agricultural routes [1]. One example of secondary dispersion throughout northern Europe involves a group of sheep breeds called the north European short-tailed sheep. These sheep were spread by Norse Vikings from the eighth to the middle of the eleventh century and can now be found in a range from Russia to Iceland [3]. Phenotypically, many of the north European short-tailed breeds are characterized by their primitive phenotypes, variation in coat colour, presence of horns and short fluke shaped tails [3]. Genetically, north European short-tailed sheep have been shown to be genetically unique, with some breeds contributing more than expected to overall domestic sheep diversity, although many of these populations have a small effective population size and low within breed diversity [4].

Many of the north European short-tailed sheep breeds today are local breeds, having adapted to specific environments, and in Sweden these small local populations are recorded and maintained as separate heritage breeds [3]. Genetic diversity has only been studied in some of these breeds: microsatellite markers were used for comparing north European short-tailed sheep [4] and northern Eurasian breeds [5]; population history has been studied in native Swedish breeds using endogenous retroviruses to compare genotypes with Texel and British breeds [6, 7]; and seven microsatellite markers and pedigree data were used to estimate inbreeding and genetic diversity in a local Swedish breed, Gute sheep [8].

Some north European short tailed sheep breeds were included in the Sheep HapMap project [9] where many breeds from around the world were genotyped using a medium-density SNP array [10]. However, native Swedish breeds have not yet been genotyped at medium- or high-density and have not been included in comparisons with breeds from around the world and were not part of the Sheep HapMap project. These comparisons and studies using a higher density of DNA markers will be important for informing future discussions on the conservation of these breeds.

Our objectives were to study population structure of native Swedish sheep breeds using the 600 K SNP array and to analyse the structure of European and southwestern Asian populations, including native Swedish sheep populations, using data from the 600 K SNP array.

## Results

For the genetic diversity measures, Gotland sheep, the commercial breed in these analyses, had the highest number, but not the longest ROH, and had fairly low inbreeding estimates. In contrast the Fjällnäs sheep, the local breed with the smallest population size, had the longest ROH on average and large inbreeding estimates. All genetic diversity measures estimated for the five native Swedish sheep breeds are in Table 1 including three inbreeding measures and expected and observed heterozygosity. Supplementary Figures 2 and 3 show the linkage disequilibrium decay and the minor allele frequencies of the 600 K SNPs genotyped in this study for the five Swedish breeds. Supplementary Figure 4 shows the distribution of length of ROH.

**Table 1:** Genetic diversity measures for five native Swedish sheep breeds

Breed	N	Average number of ROH/individual	Average length of ROH (kb)	Average Fhat1	Average Fhat2	Average Fhat3	Observed heterozygosity (%)	Expected heterozygosity (%)
Dalapäls	21	342.62	2747.95	0.18	0.30	0.24	30.29	31.12
Fjällnäs	10	333.00	3638.23	0.60	0.36	0.48	29.98	35.93
Gotland	19	504.42	1019.77	0.25	0.03	0.14	33.45	33.35
Gute	22	478.45	1677.55	0.13	0.25	0.19	31.44	32.06
Klövsjö	21	309.62	2753.31	0.18	0.26	0.22	33.55	33.89

**Fig. 1:**
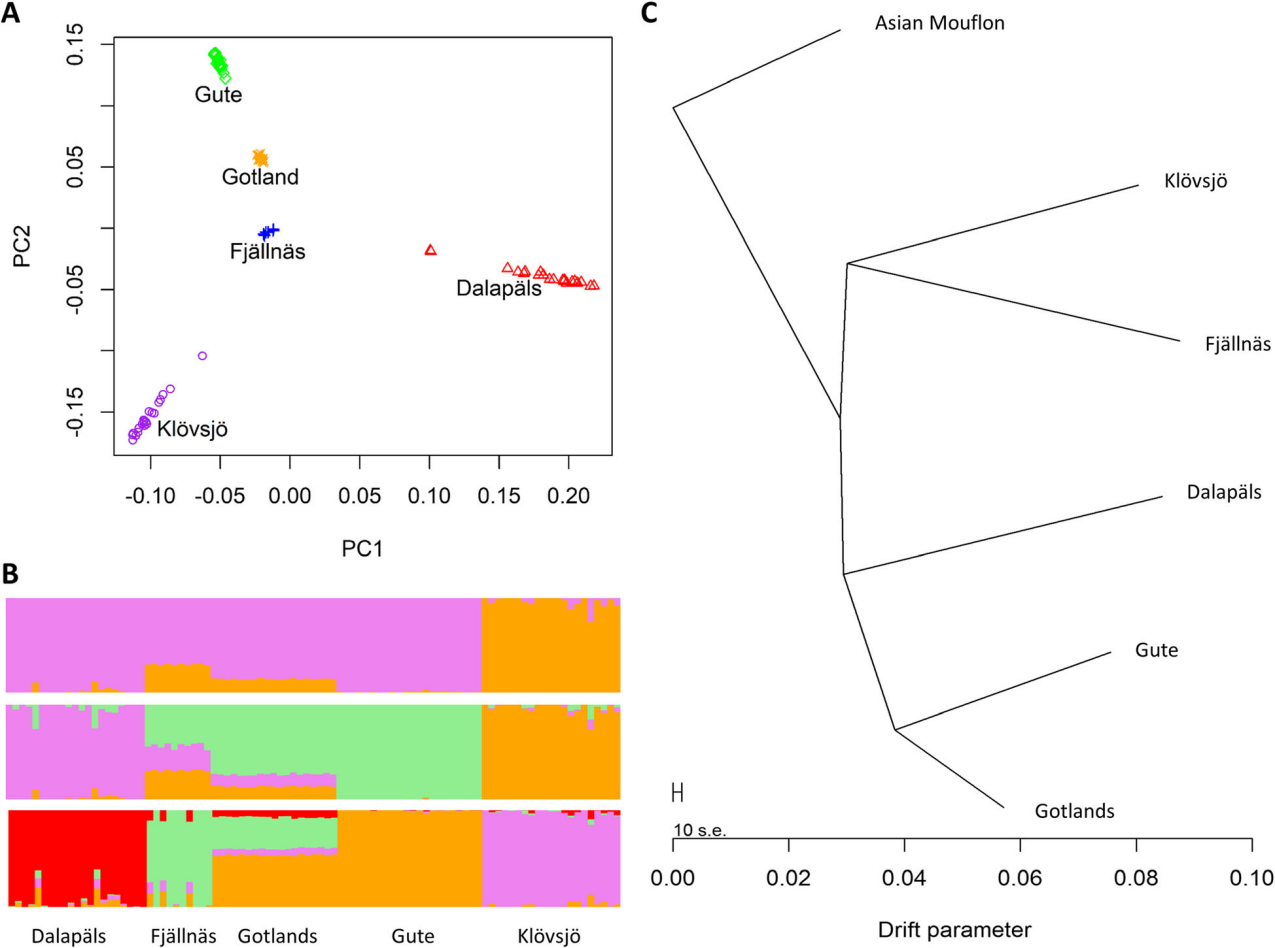
Population structure in five Swedish breeds: **a**. Principal component analysis, **b**. individual ancestry coefficients for two (top), three (middle) and four (bottom) ancestral populations, and **c**. a maximum likelihood population tree (rooted with Asian Mouflon sheep)

The principal component analysis (PCA) showed that all five Swedish breeds were distinguishable from one another and the individuals within each breed clustered close together (with the exception of two Dalapäls individuals that were some distance from the remaining individuals) using the first two principal components (Fig. 1a). When individual ancestry coefficients were estimated for two, three and four (which is the optimal number based on cross-entropy criterion) ancestral populations (Fig. 1b), Klövsjö was the most distinct breed followed by Dalapäls. Gotland sheep were shown to be more related to Gute sheep and there appeared to be some shared ancestry between Fjällnäs and Gotland sheep. Two of the Dalapäls sheep were different from other Dalapäls sheep (they are the two outliers shown in Fig. 1a) and three Fjällnäs sheep were different from other Fjällnäs sheep. In the population tree rooted by the Asian mouflon sheep (Fig. 1c) the five breeds were distinct and had long branch lengths indicating high amounts of genetic drift. Gute and Gotland sheep had a closer relationship than the other breeds: they both were on the same branch although they also had long branch lengths.

**Fig. 2:**
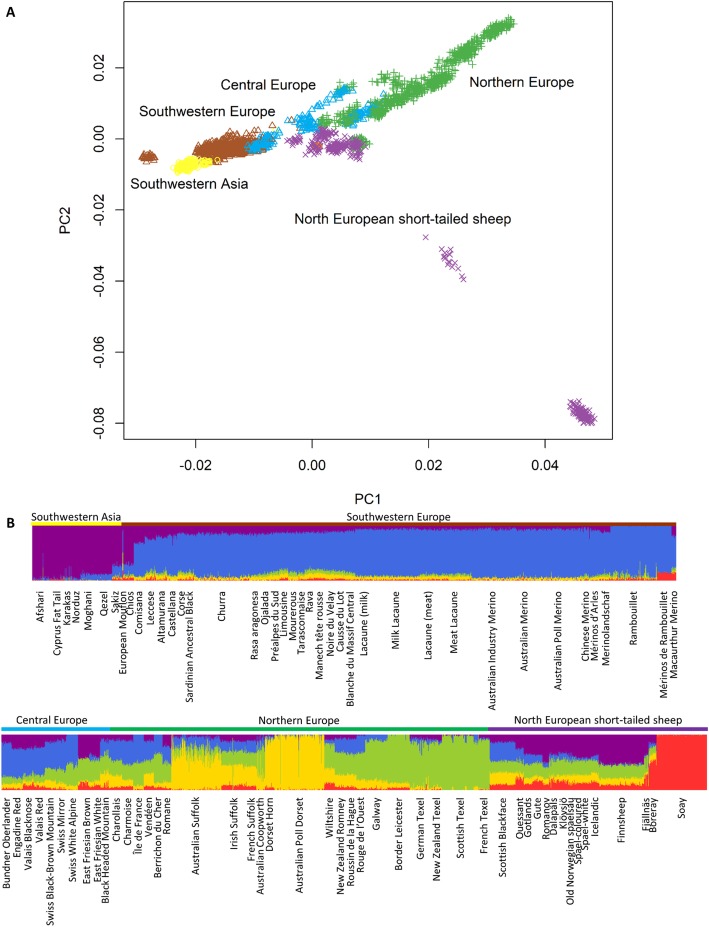
Population structure for southwestern Asian and European sheep breeds from SNP genotypes using **a**. Principal component analysis to study population structure, and **b**. individual ancestry coefficients for five ancestral populations. European Mouflon samples are represented as red diamonds

**Fig. 3:**
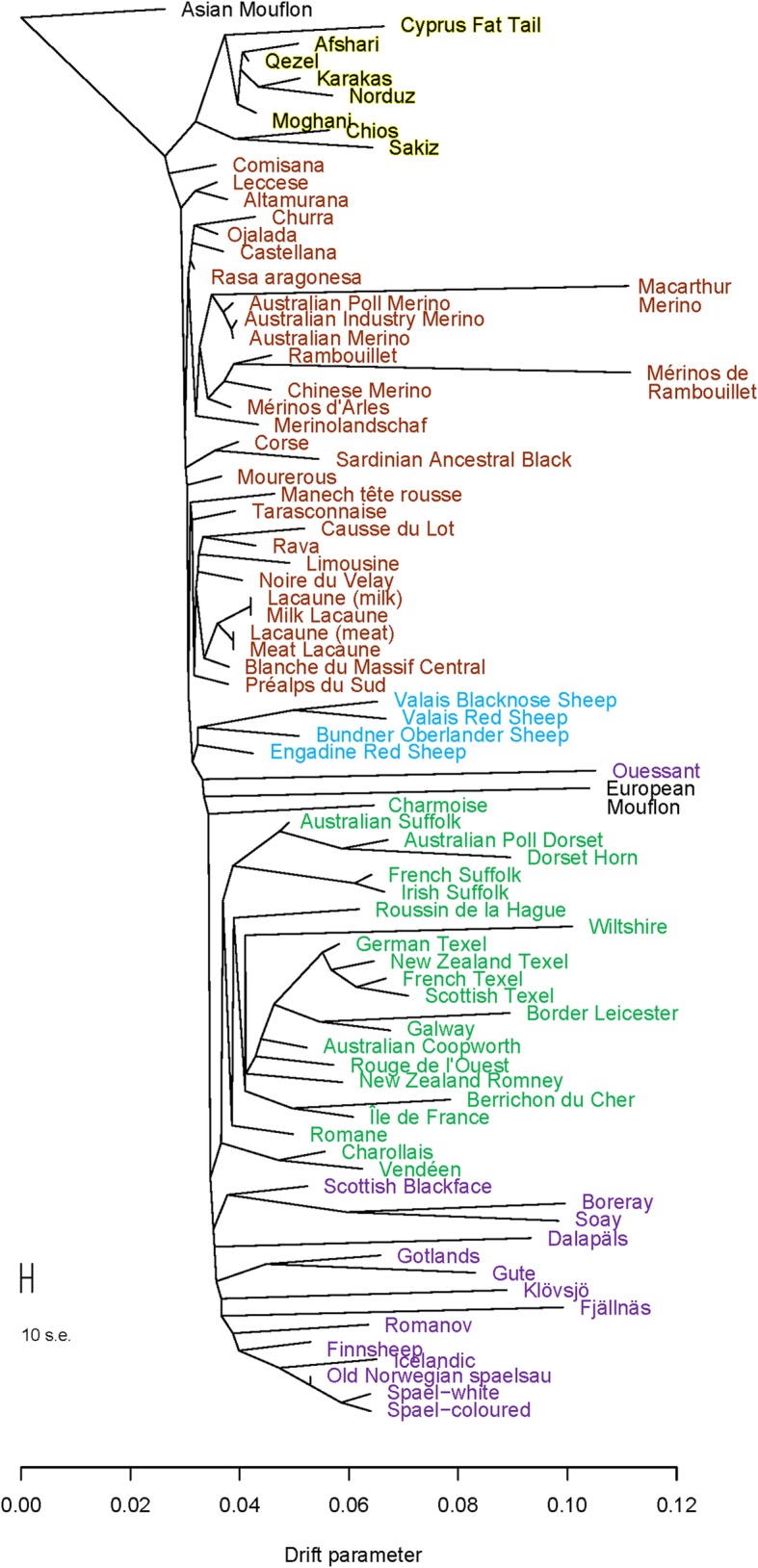
Population structure for southwestern Asian and European sheep breeds from SNP genotypes: maximum likelihood population tree (rooted with Asian Mouflon, southwestern Asian breeds in gold, southwestern European breeds in brown, central European breeds in blue, northern European breeds in green and north European short-tailed breeds in purple)

When we included the other European and southwestern Asian breeds in the PCA, breeds generally grouped with others from the same geographical area of origin (Fig. 2a). There were two exceptions to this: there was one breed that did not group closely with other southwestern European breeds, the Mérinos de Rambouillet (the furthest left brown cluster of the PC plot); and there were two north European short-tailed breeds which were quite distinct from all other breeds, the Soay (the purple cluster at the bottom of the PC plot) and Boreray (the purple cluster second from the bottom of the PC plot) sheep breeds. The Swedish sheep breeds grouped with the remaining north European short-tailed sheep included in this analysis. Breeds continued to group by geographical origin when looking at the individual ancestry coefficients (Fig. 2b). The population tree demonstrated a more detailed look at the genetic relationships between European and Asian breeds (Fig. 3). Swedish breeds grouped with the other north European short-tailed sheep and this group generally had long branch lengths in comparison with some other geographic groups (ex. Southwestern Asian sheep have very short branch lengths).

**Fig. 4:**
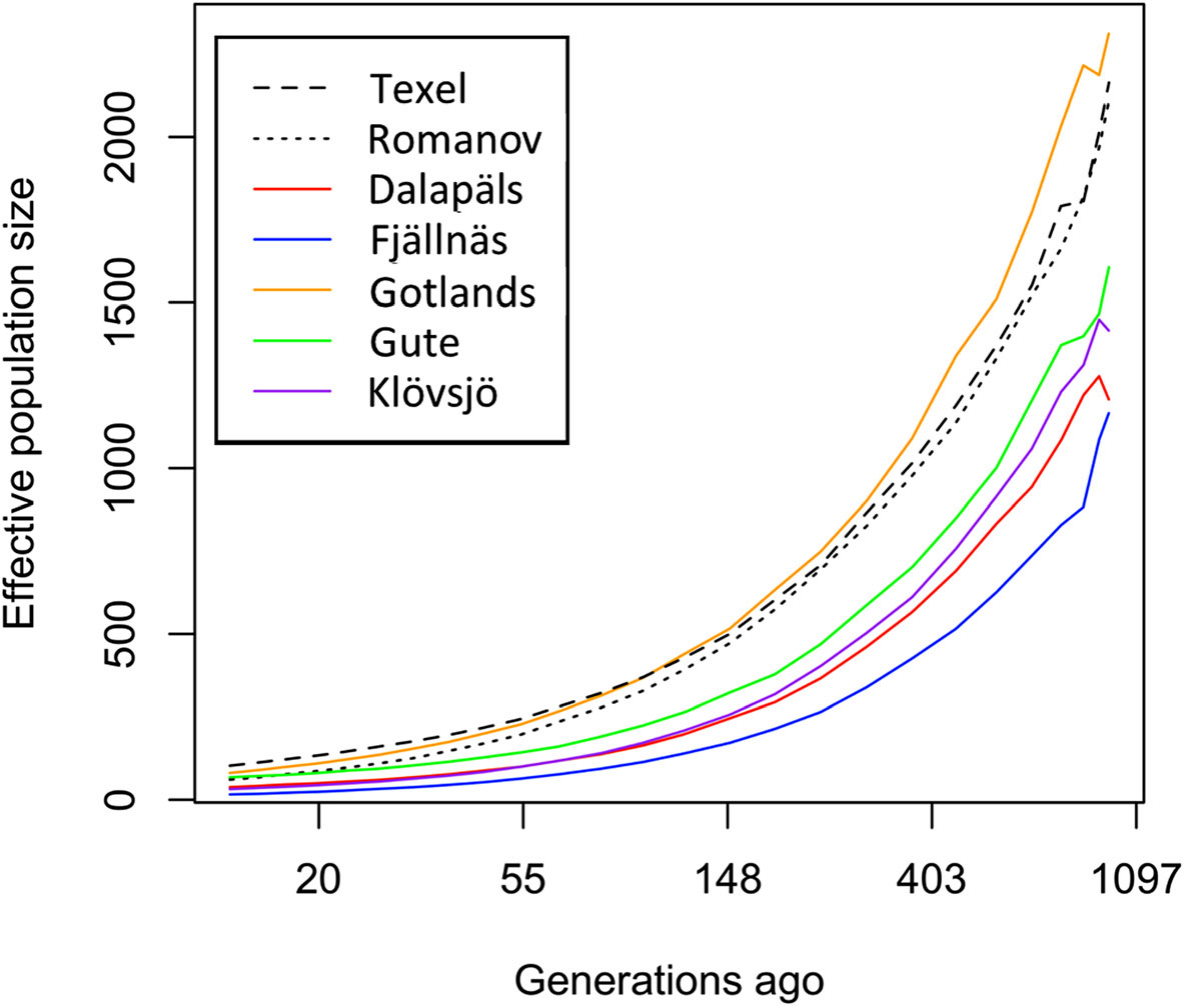
Estimates of historic effective population size for five native Swedish breeds (Dalapäls, Fjällnäs, Gotland, Gute, Klövsjö) and two French populations (Texel and Romanov)

Estimates of historic effective population size are presented in Fig. 4. The four local Swedish sheep breeds (Dalapäls, Fjällnäs, Gute, and Klövsjö sheep) are consistently smaller in size over time compared to commercial breeds Gotland, Texel and Romanov populations. The most recent estimates of effective population size (13 generations ago, approximately 40 years ago) were: Dalapäls, 38; Fjällnäs, 16; Gotland, 81; Gute, 68; Klövsjö, 32; Texel, 103; Romanov, 61.

## Discussion

Dalapäls sheep, Klövsjö and Fjällnäs sheep have similar numbers of ROH per individual, but the average length of ROH is clearly larger in Fjällnäs sheep than in the other breeds. This can be explained by the fact that the samples from Fjällnäs were collected shortly after the breed was discovered and the samples originate from two farms where the sheep had been isolated with small population sizes. Thus, the mating of close individuals has likely taken place to a large extent in Fjällnäs sheep. This is in agreement with the lowest observed heterozygosity in Fjällnäs sheep among the studied Swedish sheep breeds. It is interesting that Gute sheep had smaller ROH than the other local sheep breeds. It corresponds well with the fact that they have a larger current population size, but also with the previous result that the inbreeding in Gute sheep is rather low [8]. The LD is very high in Fjällnäs sheep and lowest in Gotland sheep. This is in agreement with the population sizes where Fjällnäs is a very small population and Gotland has the largest population. Also the Fjällnäs sheep was recently founded by a combination of two small herds that had a common origin but had been isolated from each other for some time. The two herds might have had differences in allele frequency at many loci due to genetic drift which would lead to high LD the first generations after the herds were combined again.

We used principal component analysis (PCA), estimated individual ancestry coefficients and estimated population trees to study the population structure of five Swedish sheep breeds and to compare the population with other European and southwestern Asian breeds.

Different strategies have been employed to preserve north European short-tailed sheep breeds [3]: in Sweden, small local sheep populations are maintained through the Föreningen Svenska Allmogefår (a society currently maintaining 10 local breeds (http://allmogefar.se/)). Local Swedish breeds were defined based on the phenotypes and geographic area that they have adapted and traditionally confined to. It is an interesting exercise to study population structure in these breeds to estimate their relationships with one another and to see if they are in fact separate breeds. Tapio et al. [4] showed that Dalapäls and Gute sheep were unique and Gotland sheep were related to Gute sheep. Mukiibi et al. [6] showed that while Gute and Klövsjö were somewhat related and shared two retrotypes (including R0 which is a characteristic of primitive breeds), Gute had four retrotypes and Klövsjö had one retrotype not shared with the other breeds [6]. Our results from all three analyses (principal components analysis, mixture model and population tree model) were complimentary to these previous results: all five breeds are separate breeds, even though Gute and Gotland sheep breeds were related. The Fjällnäs were of particular interest as this was the first time this breed was studied using high-density genotyping. We included all (*N* = 10) of the adult sheep in this population from 2011, the year this breed was officially recognized. Our results showed that these animals grouped together based on their genotypes as seen in the close clustering of these individuals in the PCA and were not close to other breeds in the population tree. However, we detected at least two groups in this breed of only 10 individuals, which is probably because the official breed formation occurred only recently by combining a small number of flocks. Animals from the same breeds generally grouped close together in the PCA and the ancestry coefficient analysis were consistent with some exceptions including two Dalapäls sheep. These two individuals are not closely related to each other and are not likely to be the result of unintentional crossbreeding as owners have not kept rams from other breeds. The two Dalapäls sheep were born in 2005 and 2006 and their ancestors had long generation intervals which might be why they are genetically different from the other Dalapäls sheep genotyped in this study: there are very few generations between them and founding individuals of the breed in comparison. There are more generations between other Dalapäls sheep and their ancestors and therefore more opportunity for selection and drift.

When we studied the population structure including data from southwestern Asian and European breeds, we saw that population structure in sheep was related to geography. This was in agreement with the previously published study by Kijas et al. [11]. Two breeds were very different from others in both the PCA and when estimating ancestry coefficients: Boreray and Soay sheep. These two breeds are small isolated populations that have been subjected to a lot of drift which explains why they were so different in the first two analyses. However, these two breeds do group with other north European short-tailed sheep in our analyses.

In the population tree, in general, there were very short branch lengths in southwestern Asian and southwestern European sheep breeds, longer branch lengths in central and northern European sheep, and very long branches in north European short-tailed sheep, including Swedish sheep breeds. Livestock breeds further from domestication centres tend to have less genetic diversity [5, 12, 13] and branch length (amount of drift) is an indicator of this reduced within breed genetic diversity in this study.

The estimated historic effective population size for Gute sheep was smaller than previous estimates (68 compared with 83 and 155 [8]). These three estimates are from different data sets: 68 was estimated in this study using high density genotyping of 22 animals, while 83 was estimated from microsatellite genotypes of 96 individuals and 155 was estimated using pedigree information from the entire registered population of Swedish Gute sheep. The estimate from the pedigree is likely an overestimate as this estimate is affected by the depth of the recorded pedigree. While the estimates from microsatellite and high-density SNP genotyping are likely more reflective of the effective population size of this small population.

The breeds raised for commercial purposes, Gotland, Texel and Romanov, had higher historic effective population sizes. This could be because of historic crossbreeding. Although Gotland sheep have shared ancestors with Gute (relatively recently), Gotland sheep are believed to have been crossed with other breeds including Romanov sheep to improve certain traits. The populations of Texel and Romanov used in this study were from France and not their country of origin and crossbreeding over time cannot be overlooked for these populations. The other four populations of sheep, Dalapäls, Fjällnäs, Gute, and Klövsjö, grouped together and their estimated effective population sizes were reflective of their current population sizes.

There have been other estimates of effective population size in sheep including local Swiss breeds [14, 15], local Danish breeds [16] and local French breeds [17]. Effective population size estimates (from pedigree) of Danish breeds, Marsk (Ne = 23) and Såne (Ne = 22) sheep [16], were similar to estimates of Swedish local breeds. In contrast, just one Swiss local breed, Valais Red sheep (Ne = 95), had a similar historical effective population estimate (29 generations ago, estimated using 50 K SNP) [14, 15] to Gute sheep, one of the larger populations of local Swedish breeds. In addition, two populations of Romanov sheep (part of the north European short tailed sheep group) raised in France were similar in size to local Swedish breeds (Ne = 78 and 65, estimated using pedigree) [14, 15]. These low numbers were also seen in other local short-tailed breeds raised in other countries (Denmark: Finnsheep (Ne = 36), Spel (Ne = 40) and Icelandic sheep (Ne = 30)) [16]. With these comparisons, Swedish local breeds generally have low effective population sizes compared with other north European short-tailed sheep and other local northern European sheep breeds. When comparing our results with estimates from the Sheep HapMap project, breeds had much higher estimates of effective population size using 50 K SNP (Finnsheep (Ne = 795), Soay (Ne = 194), white Spael (Ne = 339), Scottish Blackface (Ne = 528), Scottish Texel (Ne = 305), German Texel (Ne = 448) and New Zealand Texel (Ne = 282)) [11]. The relatively low effective population size of local Swedish breeds adds to the evidence that Swedish breeds on average have been subjected to more genetic drift than other breeds.

## Conclusions

This is the first time that the population structure of native Swedish sheep breeds has been described using high density SNP data. The five native Swedish breeds included were separate and generally unique breeds. We were also able to compare the population structure of the Swedish breeds with other European and southwestern Asian breeds using information from 40 K markers. Breeds grouped by geographic origin. We could show for the first time that Swedish sheep grouped with other north European short-tailed sheep breeds from Iceland, France, Scotland, Norway, Finland and Russia.

## Methods

The Swedish short-tailed sheep populations represented in this study were: Dalapäls, Fjällnäs, Gotland, Gute, and Klövsjö sheep breeds. These five breeds represented different geographic origins within Sweden and different population sizes. Locations of the origins of breeds have been included in Supplementary Figure 1 and population information on registered individuals in Sweden have been included in Supplementary Table1.

Dalapäls (also known in English as Dala fur or Dala pelt) sheep are from Dalarna (a county located in the middle of Sweden that shares a border with Norway) and have been bred for their skin which was used in traditional costumes. Their wool is curly and finer than that of most other local Swedish breeds. These sheep are usually white and males are horned while females are usually polled.

Accepted officially as a breed in 2011, Fjällnäs sheep are currently found in Norrbotten, a county bordering with Finland. Individuals are light fawn in colour and males have small brittle horns while females are polled. The breed has only 10 adults in 2011 and all of these individuals were genotyped and included in this study.

Gotland and Gute sheep originate from the same population of sheep on the island/county of Gotland in the Baltic Sea. Polled sheep became popular and eventually became the Gotland breed and in order to preserve the primitive sheep, the remaining horned individuals were gathered in the 1920s to become Gute sheep breed. Today Gute sheep are bred for conservation purposes while the Gotland sheep are bred for improved pelt quality (colour and curl of the fibre). Many Gotland and Gute sheep are some shade of grey with the Gotland sheep fleece more uniform in colour. Gotland males and females are polled while Gute males and females are horned.

Klövsjö sheep are from the surrounding area of a village, Klövsjö, in Jämtland (a county north of Dalarna, also sharing a border with Norway). These sheep are usually black or white and both sexes are usually polled.

Blood samples were collected from native Swedish sheep. Ethical permission was obtained prior to the collection of blood samples. Genomic DNA used in this study was extracted from fresh blood samples using a QIAsymphony® DSP DNA extraction robot and DSP DNA Midi Kit (QIAGEN). For this study 21 Dalapäls, 10 Fjällnäs, 19 Gotland, 22 Gute and 21 Klövsjö sheep were genotyped for 653,305 autosomal SNPs (Ovine Infinium® HD SNP BeadChip) at Labogena, INRA, Paris, France. Of the 22 Gute sheep genotyped: 19 sheep were genotyped for seven microsatellite markers and included in a study by Rochus and Johansson [8]; and eight sheep had retroviruses and microsatellites genotyped and were included in both studies by Mukiibi et al. [6] and Rochus and Johansson [8]. Of the 21 Klövsjö sheep included in this study, three sheep had retroviruses genotyped and were included in Mukiibi et al. [6].

Quality control and filtering of SNPs included removing SNPs with a minor allele frequency of zero, removing SNPs with a missing rate greater than 0.01 and removing sheep with a missing genotype rate greater than 0.15 using PLINK 1.9 [18, 19]. All of the 93 sheep, and a total of 502,144 SNPs passed quality control and filtering steps.

Genetic diversity measures were estimated using PLINK 1.9 [18, 19] including the number and size of runs of homozygosity (ROH) (using the following settings: max inverse density (kb/SNP) = 1000, minimum length = 100, minimum SNP count = 20, scanning window size = 20, maximum heterozygotes in a scanning window = 1), and three inbreeding estimates: Fhat1 (variance-standardized relationship minus 1), Fhat2 (heterozygosity), and Fhat3 (based on the correlation between uniting gametes).

Three methods were used to study population structure in the five Swedish breeds: principal components analysis using the software PLINK 1.9 [18, 19], a mixture model to estimate individual ancestry coefficients estimated using the software sNMF (R version 2.0) [20] (using default settings) and a population tree model which also estimates mixture events determined using the software Treemix 1.13 [21] (grouping SNPs by 100 and using the Asian Mouflon as the tree root). For the population tree model we included SNP frequencies from Asian Mouflon sequences, which was data generated by the NEXTGEN consortium (http://nextgen.epfl.ch/).

We then combined our dataset of Swedish breeds with a French sheep dataset (also genotyped with Ovine Infinium® HD SNP BeadChip, first presented in a study by Rochus et al. [22] and available from https://zenodo.org/record/237116) and European and southwestern Asian sheep (genotyped for 49,034 SNP first presented in [10] available from http://www.sheephapmap.org). Only the SNPs shared between all datasets were used to study population structure using principal components analysis [18, 19], estimating individual ancestry coefficients [20] and estimating a population tree [21]. We included 38,589 SNPs and 2938 sheep from 87 populations. For the population tree model, we again included the SNP frequencies from Asian Mouflon sequences to root the tree.

We estimated the historic effective population sizes of the five native Swedish breeds and two French populations (Texel and Romanov). We included the two French populations for comparative purposes. Texel sheep are a common breed originating from the Netherlands but is bred all around the world today for commercial meat production. Romanov sheep are a north European short-tailed sheep breed that originated in Russia. This breed is also used more widely. We estimated historic effective population size using the default settings (MAF ≥0.05, minimum distance between SNPs: 50 kb, maximum distance between SNPs: 4000 kb, and ≤ 100,000 SNPs for each chromosome) using the software SNeP 1.1 [14].

## Supplementary information

**Additional file 1: Figure S1.** Map of the origin of Swedish local breeds.

**Additional file 2: Figure S2.** Linkage disequillibrium decay for 600 K SNP genotyped in five native Swedish breeds (Dalapäls (DAL), Fjällnäs (FJA), Gotland (GOT), Gute (GUT), Klövsjö (KLO)).

**Additional file 3: Figure S3.** Minor allele frequency of 600 K SNPs genotyped in Dalapäls, Fjällnäs, Gotland, Gute, and Klövsjö sheep.

**Additional file 4: Figure S4.** Distribution of length of ROH calculated from 600 K SNPs genotyped in Dalapäls, Fjällnäs, Gotland, Gute, and Klövsjö sheep.

**Additional file 5: Table S1.** Purebred Swedish sheep registered in Elitlamm (2018).

## Data Availability

The datasets used and/or analysed during the current study are available from the corresponding author on reasonable request and will be publicly available on the Dryad digital repository (https://datadryad.org/) once the manuscript is published.

## Abbreviations

Fhat1: Inbreeding coefficient (variance-standardized relationship minus one)
Fhat2: Inbreeding coefficient (approximately equal to (observed homozygous count – expected count) / (total observations – expected count))
Fhat3: Inbreeding coefficient (based on the correlation between uniting gametes)
MAF: Minor allele frequency
Ne: Effective population size
PCA: Principal component analysis
ROH: Runs of homozygosity
