# Supplementary figures and images for: Population structure of five native sheep breeds of Sweden estimated with high density SNP genotypes
Source: BMC Genet. 2020 Mar 6;21:27. doi: 10.1186/s12863-020-0827-8 (PMC7060653; doi:10.1186/s12863-020-0827-8)

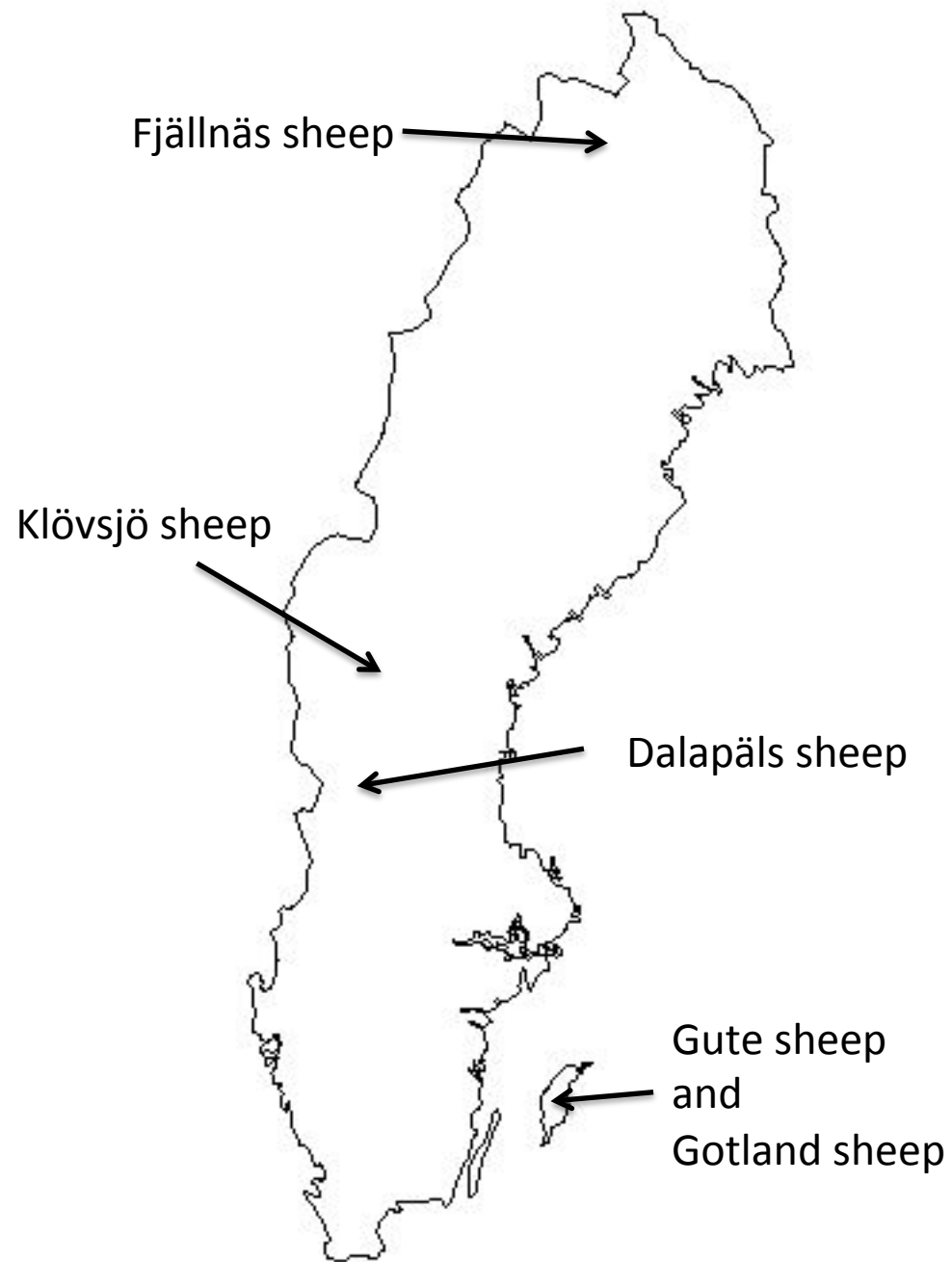

Supplement: Supplementary file 1 — Additional file 1: Figure S1. Map of the origin of Swedish local breeds. [file 12863_2020_827_MOESM1_ESM.pdf]

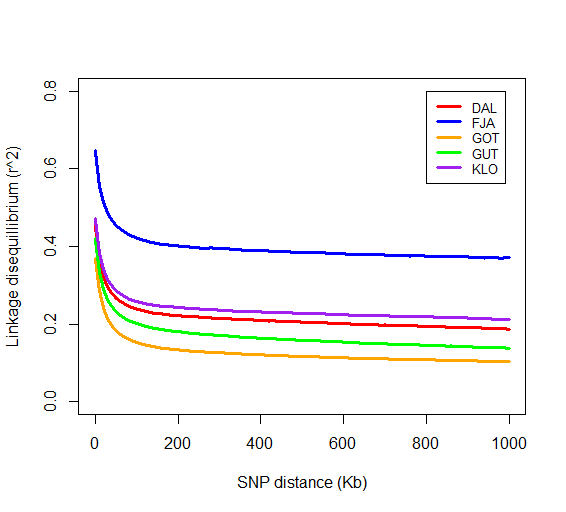

Supplement: Supplementary file 2 — Additional file 2: Figure S2. Linkage disequillibrium decay for 600 K SNP genotyped in five native Swedish breeds (Dalapäls (DAL), Fjällnäs (FJA), Gotland (GOT), Gute (GUT), Klövsjö (KLO)). [file 12863_2020_827_MOESM2_ESM.tiff]

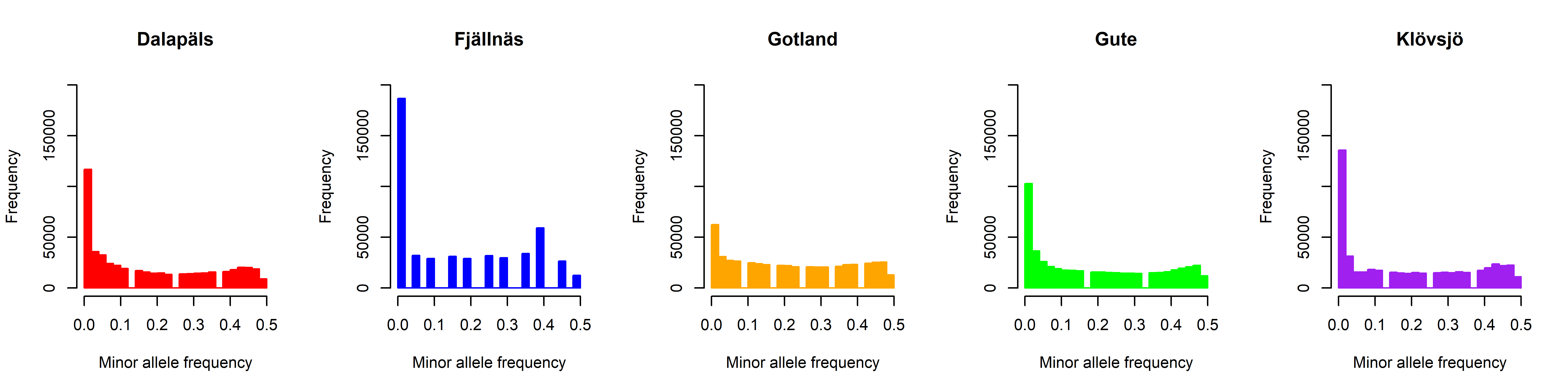

Supplement: Supplementary file 3 — Additional file 3: Figure S3. Minor allele frequency of 600 K SNPs genotyped in Dalapäls, Fjällnäs, Gotland, Gute, and Klövsjö sheep. [file 12863_2020_827_MOESM3_ESM.tiff]

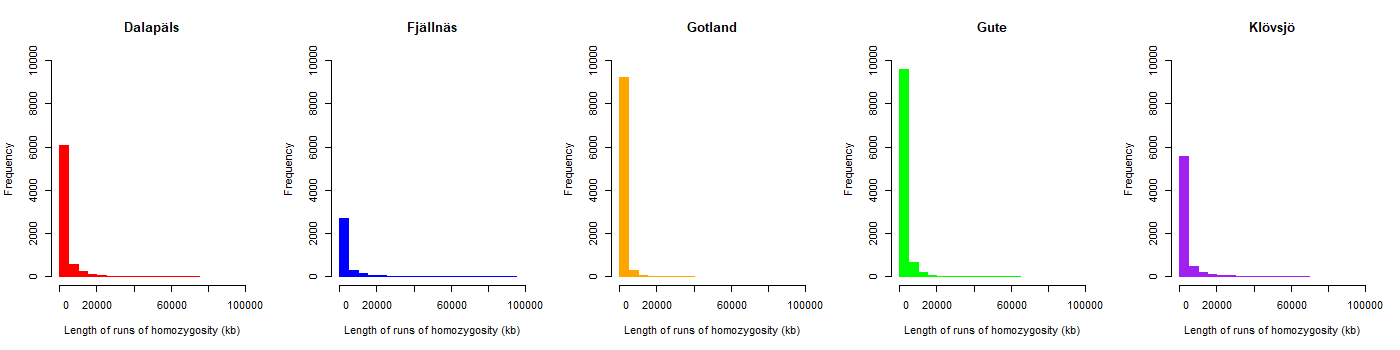

Supplement: Supplementary file 4 — Additional file 4: Figure S4. Distribution of length of ROH calculated from 600 K SNPs genotyped in Dalapäls, Fjällnäs, Gotland, Gute, and Klövsjö sheep. [file 12863_2020_827_MOESM4_ESM.tiff]
