# Supplementary material for: Population structure of five native sheep breeds of Sweden estimated with high density SNP genotypes
Source: BMC Genet. 2020 Mar 6;21:27. doi: 10.1186/s12863-020-0827-8 (PMC7060653; doi:10.1186/s12863-020-0827-8)
Supplement: Supplementary file 5 — Additional file 5: Table S1. Purebred Swedish sheep registered in Elitlamm (2018). [file 12863_2020_827_MOESM5_ESM.docx]

Supplementary Table 1: Purebred Swedish sheep registered in Elitlamm (2018)^1^

|  | Dalapäls | Fjällnäs | Klövsjö | Gotland | Gute |
| --- | --- | --- | --- | --- | --- |
| Number of herds | 27 | 2 | 69 | 520 | 107 |
| Number of ewes mated within breed | 158 | 12 | 457 | 16388 | 1342 |
| Number of ewes total with lambs 2018 | 170 | 12 | 489 | 17512 | 1438 |
| Number of rams mated within breed | 31 | 72 | 2 | 984 | 138 |
| Number of rams total with lambs 2018 | 31 | 74 | 3 | 1020 | 141 |
| Number of purebred lambs born 2018 | 252 | 19 | 939 | 30926 | 2173 |

^1^ Data from: <https://www.elitlamm.com/pub/default.aspx?section=Svenska+F%C3%A5ravelsf%C3%B6rbundet>
